# Marine Invasion in the Mediterranean Sea: The Role of Abiotic Factors When There Is No Biological Resistance

**DOI:** 10.1371/journal.pone.0031135

**Published:** 2012-02-21

**Authors:** Emma Cebrian, Conxi Rodríguez-Prieto

**Affiliations:** 1 Departament de Ciències Ambientals, Facultat de Ciències, Universitat de Girona, Girona, Spain; 2 Centre d'Estudis Avançats de Blanes (CSIC), Blanes, Spain; Lakehead University, Canada

## Abstract

The tropical red alga *Womersleyella setacea* (Rhodomelaceae, Rhodophyta) is causing increasing concern in the Mediterranean Sea because of its invasive behavior. After its introduction it has colonized most Mediterranean areas, but the mechanism underlying its acclimatization and invasion process remains unknown. To understand this process, we decided i) to assess *in situ* the seasonal biomass and phenological patterns of populations inhabiting the Mediterranean Sea in relation to the main environmental factors, and ii) to experimentally determine if the tolerance of *W. setacea* to different light and temperature conditions can explain its colonization success, as well as its bathymetric distribution range. The bathymetric distribution, biomass, and phenology of *W. setacea* were studied at two localities, and related to irradiance and temperature values recorded *in situ*. Laboratory experiments were set up to study survival, growth and reproduction under contrasting light and temperature conditions in the short, mid, and long term.Results showed that, in the studied area, the bathymetric distribution of *W. setacea* is restricted to a depth belt between 25 and 40 m deep, reaching maximum biomass values (126 g dw m^−2^) at 30 m depth. In concordance, although in the short term *W. setacea* survived and grew in a large range of environmental conditions, its life requirements for the mid and long term were dim light levels and low temperatures. Biomass of *Womersleyella setacea* did not show any clear seasonal pattern, though minimum values were reported in spring. Reproductive structures were always absent. Bearing in mind that no herbivores feed on *Womersleyella setacea* and that its thermal preferences are more characteristic of temperate than of tropical seaweeds, low light (50 µmol photon m^−2^ s^−1^) and low temperature (12°C) levels are critical for *W. setacea* survival and growth, thus probably determining its spread and bathymetric distribution across the Mediterranean Sea.

## Introduction

The spread of non-indigenous species is claimed to cause dramatic ecological impacts and is considered a major threat for biodiversity conservation [1]. The Mediterranean Sea is one of the areas of the world most severely hit by those impacts with about 955 introduced species [2], among which macroalgae are considered to be especially worrying [3] because they may alter both ecosystem structure and function by monopolizing space and developing into ecosystem engineers [4]. The red alga *Womersleyella setacea* (Hollenberg) R.E. Norris is one of at least eight species that can be assigned to the category of invasive macroalgae in the Mediterranean [3]. In many Mediterranean localities it is exceedingly abundant, forming thick, persistent carpets that completely cover deep sublittoral rocky substrata [5]–[9], have substantial negative effects on native communities [7], [10]–[11], modify benthic assemblages [5], [8], [12]–[14], and outcompete key species [15]. *Womersleyella setacea* was described originally from the Hawaiian Islands [16] and later reported for other tropical localities both of the Pacific and Atlantic oceans [17]. It was first observed in Mediterranean coastal waters in the eighties in the Var region, France [18] and in Italy [19], and rapidly increased its distribution throughout Mediterranean waters: Corsica, Mediterranean coasts of Spain, the Balearic Islands, the Adriatic Sea, Malta and Greece [17]. The origin and way of introduction of this species remain unknown, but a suggested vector is ship hull fouling [20].

Only a small fraction of the many marine species introduced outside of their native range are able to invade and thrive in new habitats [21]. Studies of traits that make non-indigenous marine species invasive are essential to understanding the invasion procedure and to identify the key processes and filters that determine their success [22]. One of the first suggested determinants of the invasion process is the climate, since it sets broad limits to invader distribution and may cause introduced species to fail immediately during colonization [23]. However, environmental conditions that suit *W. setacea* are unknown. In fact, there is little published information about the phenology of the introduced Mediterranean populations (but see [5], [9], [13]), and the only previous study of its physiology is restricted to short response observations concerning a few weeks and a narrow range of light and temperature conditions [24], preventing further generalizations in the long term. Therefore, the main goal of the present paper is to describe the seasonal biomass and phenological patterns of natural *W. setacea* populations, relating them with the main environmental factors. In addition, we set up two batteries of laboratory experiments to 1) study seasonal patterns of *W. setacea* in relation to daylength, light and temperature, and 2) assess short- (1 month), mid- (3 months), and long-term (1 year) light and temperature requirements and tolerance for survival, growth and reproduction of specimens of a Mediterranean population of *W. setacea*. Further, we try to relate these results to its natural bathymetric distribution and its colonization success. And the adaptive capacity of *W. setacea* to winter environmental conditions, the most critical season in the Mediterranean Sea for tropical algae, was also investigated.

## Materials and Methods

### Study site

**Figure 1:**
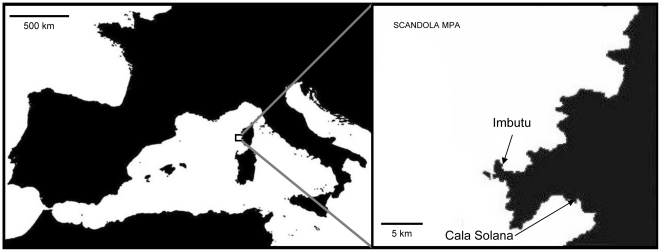
Localization of the study sites Imbutu and Cala Solana in the Scandola MPA.

The present study was carried out in the Scandola Natural Reserve (Parc Naturel Régional de la Corse), Corsica, France (Fig. 1). This marine protected area (hereafter Scandola MPA) was established in 1975 and covers 1000 ha. It has been recognized by the United Nations as a natural World Heritage Site and was inscribed in the world heritage list in 1983. The invasive species *W. setacea* was first recorded in the Scandola MPA in 1989 although it was probably introduced somewhat earlier [18], and at the beginning of this study it was already widespread in all the MPA (authors' pers. obs.).

### Bathymetric distribution of *Womersleyella setacea* in the Scandola MPA

Within the Scandola MPA two rocky bottom sites (Imbutu and Cala Solana, Fig. 1) were chosen and a perpendicular transect to shore was established at each site. Depth of both transects ranged between 0 and 40 m. Coverage of *W. setacea* was estimated by means of quadrats of 25×25 cm divided into 25 subquadrats of 5×5 cm [25]–[26], and the number of subquadrats in which *W. setacea* appeared was recorded and used as a unit of measure. Twenty quadrats (total area of 1.25 m^2^) were randomly positioned within each 5 m depth range.

### Biomass sampling procedure

**Figure 2:**
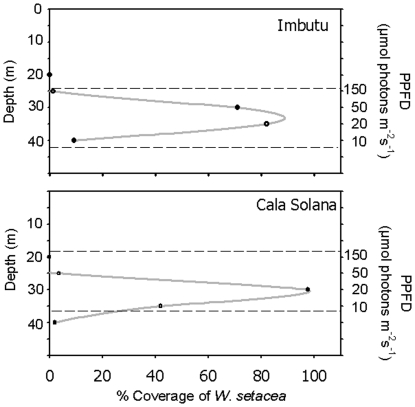
Bathymetric distribution of *W. setacea* at the two sampling localities (Imbutu and Cala Solana, Scandola MPA). Mean light (µmol photon m^−2^ s^−1^) values recorded in July at the right axes.

The study of the biomass annual cycle lasted from November 2007 to October 2008 and was performed at both sites, Imbutu and Cala Solana, at 30 m depth, in order to cope with the observed maximum *W. setacea* development (see bathymetric distribution results, Fig. 2).

At each study site three randomly positioned plots of 20×20 cm were periodically sampled throughout one year by SCUBA divers. The plots were scraped and specimens were stored in individual plastic bags. Sorting of *W. setacea* was performed in the laboratory, and its biomass was measured as dry weight (dw) by drying the sample in an oven at 60°C to constant weight.

### Photon flux density and temperature recording

To characterize light intensity in *W. setacea* distribution habitats, photon flux density was recorded at both localities by HOBO® LI data loggers (Onset Computer, EME Systems, Berkeley, CA, USA), at 25, 30, 35 and 40 m depth, hourly during one week both in summer (June) and in autumn (October).

Water temperature was recorded *in situ* in the Scandola MPA at 5, 10, 20, 25, 30, 35 and 40 m depth by Stowaway Tidbits (MicroDAQ.com, Contoocook, NH, USA) autonomous sensors (0.2°C precision, 0.15°C resolution) (see www.t-mednet.org). Temperature measures were recorded hourly during 15 months.

### Laboratory experiments

Assays were performed in laboratory culture with specimens submitted to conditions simulating the seasonal changes of daylength, light and temperature in the field ( = *seasonal experiments*), in order to confirm the seasonal pattern of *W. setacea* populations in relation to these factors. Furthermore, some specimens were submitted to constant conditions of light and temperature to study the tolerance of *W. setacea* to those factors ( = *ecophysiological experiments*).

Specimens were collected by SCUBA divers at Imbutu, on 16 October 2009, from coralligenous assemblages situated at 30 m depth. Material was placed *in situ* in opaque cotton bags to avoid high light intensities, transported in insulated boxes to the laboratory, and kept at 16°C overnight. The day after collecting, specimens were rinsed in sterilized seawater. Vegetative tufts of similar size (around 1.5 cm^2^) were exited and cultured in 250 ml vessels in incubators (Radiber, Barcelona, Spain) equipped with 30 W cool white fluorescent bulbs. Photosynthetic photon flux densities (PPFD) were measured by means of a Li-1400-501 quantometer (Li-Cor, Lincoln, NE, USA) and adjusted to the desired levels by neutral density filters. The culture medium was a quarter-strength modified Von Stosch enriched seawater medium [27] in which diatom growth was controlled by 5 mg l^−1^ GeO_2_ [28] and (cyano-) bacteria growth by 3 mg l^−1^ Penicillin-G [29]. Cultures were shaken daily by hand. The medium was changed weekly to avoid nutrient depletion, using a medium previously preheated to the experimental temperature. Specimens were cleaned of epiphytes weekly.

The survival of cultured thalli was determined as the time between the collecting date and the death of the specimen. At the beginning of the cultures (initial time), all the thalli looked rosy and healthy, but over the course of the culture the specimens went through two different survival phases. The first phase, Phase SI, began at the initial time and finished when the thalli were more than 95% damaged; and a second phase, Phase SII, began at the end of Phase SI and continued until the death of the specimens.

In seasonal experiments, cultures (with three replicates per culture) were maintained successively at daylength and temperature conditions of winter, spring, summer and autumn, combined with PPFD of 5, 10, 20, 50 and 75 µmol photon m^−2^ s^−1^ (Table 1). Changes of season in culture conditions coincided with changes of season throughout the year. In ecophysiological experiments, cultures (with four replicates per culture) were grown in a variety of temperature and PPFD regimes and 8∶16 h light∶dark (Table 2).

**Table 1:** Light, photoperiod and temperature conditions assayed in seasonal experiments.

Season	PPFD (µmol photon m^−2^ s^−1^)	Photoperiod (h light∶ dark)	Temperature (°C)
Winter	5, 10, 20, 50, 75	8∶16	12
Spring	5, 10, 20, 50, 75	12∶12	16
Summer	5, 10, 20, 50, 75	14∶10	18
Autumn	5, 10, 20, 50, 75	12∶12	16

**Table 2:** Light, photoperiod and temperature conditions assayed in ecophysiological experiments.

PPFD (µmol photon m^−2^ s^−1^)	Temperature (°C)
5, 10, 20, 50, 75	10
5, 10, 20, 50, 75	12
5, 10, 20, 50, 75	16
5, 10, 20, 50, 75	18
5, 10, 20, 50, 75	24
5, 10, 20, 50, 75	26

In both seasonal and ecophysiological experiments the length of the SI phase, the survival, and reproduction of the thalli were followed for up to one year. Cultures were monitored every week during the first three months of culture and monthly thereafter, by means of a photograph taken with a Canon EOS 350D (Canon, Tokyo, Japan). If quick changes were observed in the specimens, photographs were once again taken weekly.

The thallus surface (in mm^2^) was determined at the initial time of the culture and every two months using ImageJ (National Institutes of Health, USA). Representative specimens of material used in cultures have been deposited in the Herbarium of the University of Girona (HGI).

### Statistical analysis

*In situ* biomass was analyzed for differences between sites (Imbutu and Cala Solana) and within each site for time (five levels) by means of a Kruskal-Wallis non-parametric analysis. A non-parametric approach was used because of heteroscedasticity and departures from normality. In seasonal experiments and ecophysiological experiments a one-way ANOVA on thallus surface values obtained during the first day of the experiment was carried out to verify that all thalli undergoing different treatments started at similar conditions. In seasonal experiments, after one year of culture, final thallus surface, survival and length of Phase SI were analyzed with a one-way ANOVA with PPFD (four levels) as a fixed factor, whereas in ecophysiological experiments, thallus surface, survival and length of Phase SI were analyzed by a two-way ANOVA, where temperature (six levels) and PPFD (four levels) were fixed orthogonal factors. Tukey tests were used for *a posteriori* multiple comparisons of means data. Assumptions of normality and homogeneity of variances were examined using the Kolmogorov-Smirnov and Barlett tests, respectively. Variables were rank-transformed prior to the analysis when assumptions were not fulfilled. The analyses were performed using the STATISTICA 6.0 package.

## Results

### Bathymetric distribution

*Womersleyella setacea* displayed the highest quantitative dominance between 30 and 35 m depth, with a maximum coverage at 30 m depth, both at Imbutu and Cala Solana, while it was absent at depths shallower than 20 m (Fig. 2). *W. setacea* maximum coverage was observed at depths where incident light levels ranged between 10 and 150 µmol photon m^−2^ s^−1^, and especially between 10 and 50 µmol photon m^−2^ s^−1^ (Fig. 2).

### Biomass annual cycle

**Figure 3:**
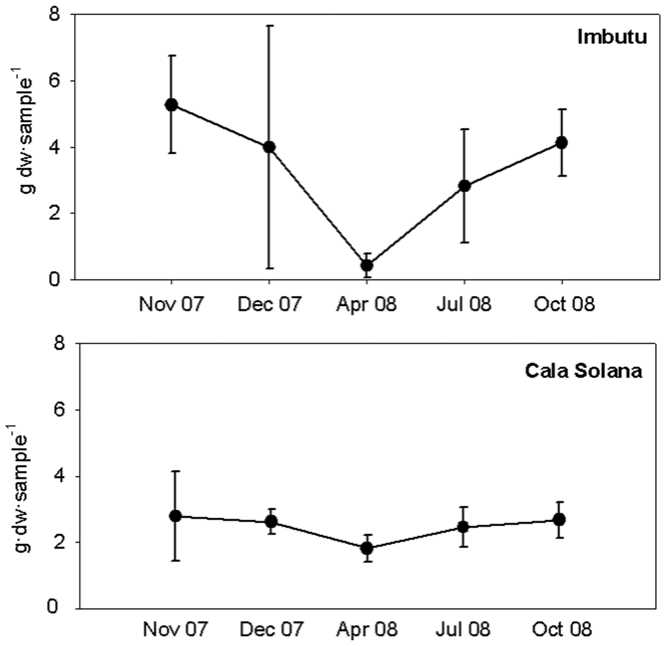
Biomass annual cycle of *Womersleyella setacea* at 30 m depth at (A) Imbutu and (B) Cala Solana. Bars represent standard deviations.

*Womersleyella setacea* biomass was similar at both localities (K-W test; p = 0.247), ranging between 0.62±0.34 (mean ±SE) g·dw·sample^−1^ on April 08 and 5.03±0.47 (mean ±SE) g·dw·sample^−1^ on November 07 at Imbutu (Fig. 3). During most of the year *W. setacea* biomass remained high and stable, without significant differences through time in Cala Solana (K-W test; p = 0.807), and only in spring *W. setacea* biomass showed a decrease in Imbutu (Tukey test p>0.05) (Fig. 3). The minimal biomass in spring is more evident at Imbutu probably because the maximal biomass is higher by comparison to Cala Solana.

### Seasonal experiments

**Figure 4:**
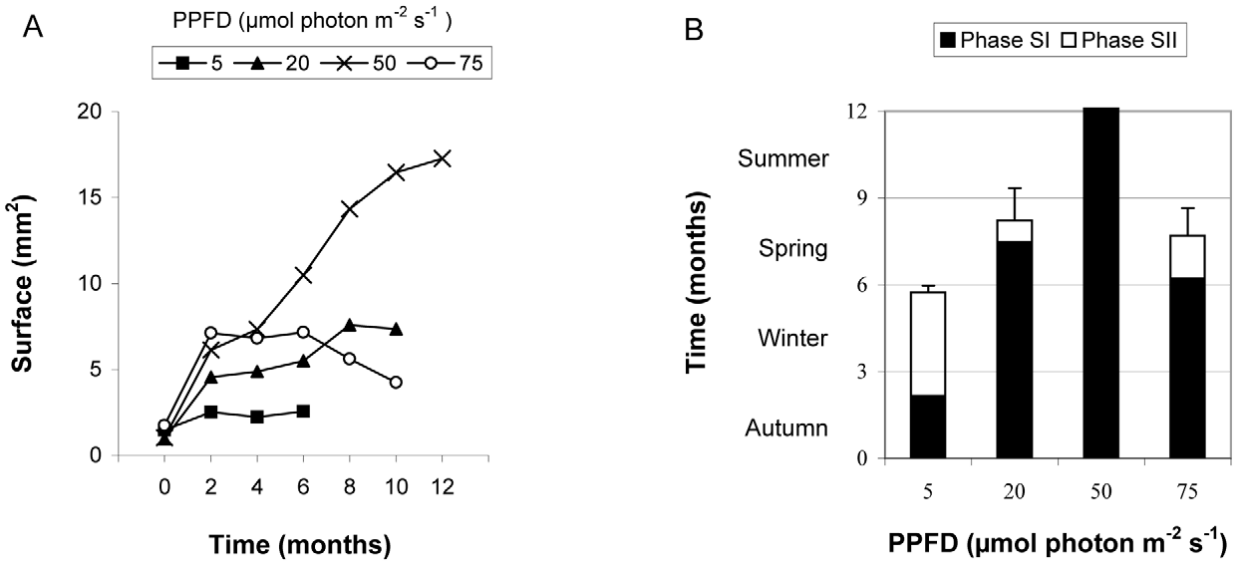
Seasonal experiments. A) Thallus surface of *W. setacea* along the culture period at the different PPFD assayed. B) Survival phases of *W. setacea* at the different PPFD assayed. Bars represent standard errors.

In seasonal experiments the surface of cultured specimens were initially similar (Fig. 4A; ANOVA-test, p>0.05), and during the experiments all specimens of all the treatments grew progressively, increasing their surface at different rates depending on the PPFD (Fig. 4A, ANOVA-test, p<0.05). Survival, and length of Phase SI, were PPFD dependent too (Fig. 4B, ANOVA-test, p<0.05), and only the specimens cultured at 50 µmol photon m^−2^ s^−1^ remained in Phase SI, looked healthy and survived all year round (Fig. 4B).The less favorable PPFD for survival and growth of *W. setacea* was 5 µmol photon m^−2^ s^−1^ where specimens survived less than six months in culture and presented the lowest thallus surface (Tukey test, p<0.05, Figs. 4A, 4B). Finally, survival and final thallus surface were similar at 20 and 75 µmol photon m^−2^ s^−1^ (Tukey test, p = 0.0741), higher than at 5 µmol photon m^−2^ s^−1^, and lower than at 50 µmol photon m^−2^ s^−1^ (Tukey test, p<0.05, Figs. 4A, 4B).

### Ecophysiological experiments

**Figure 5:**
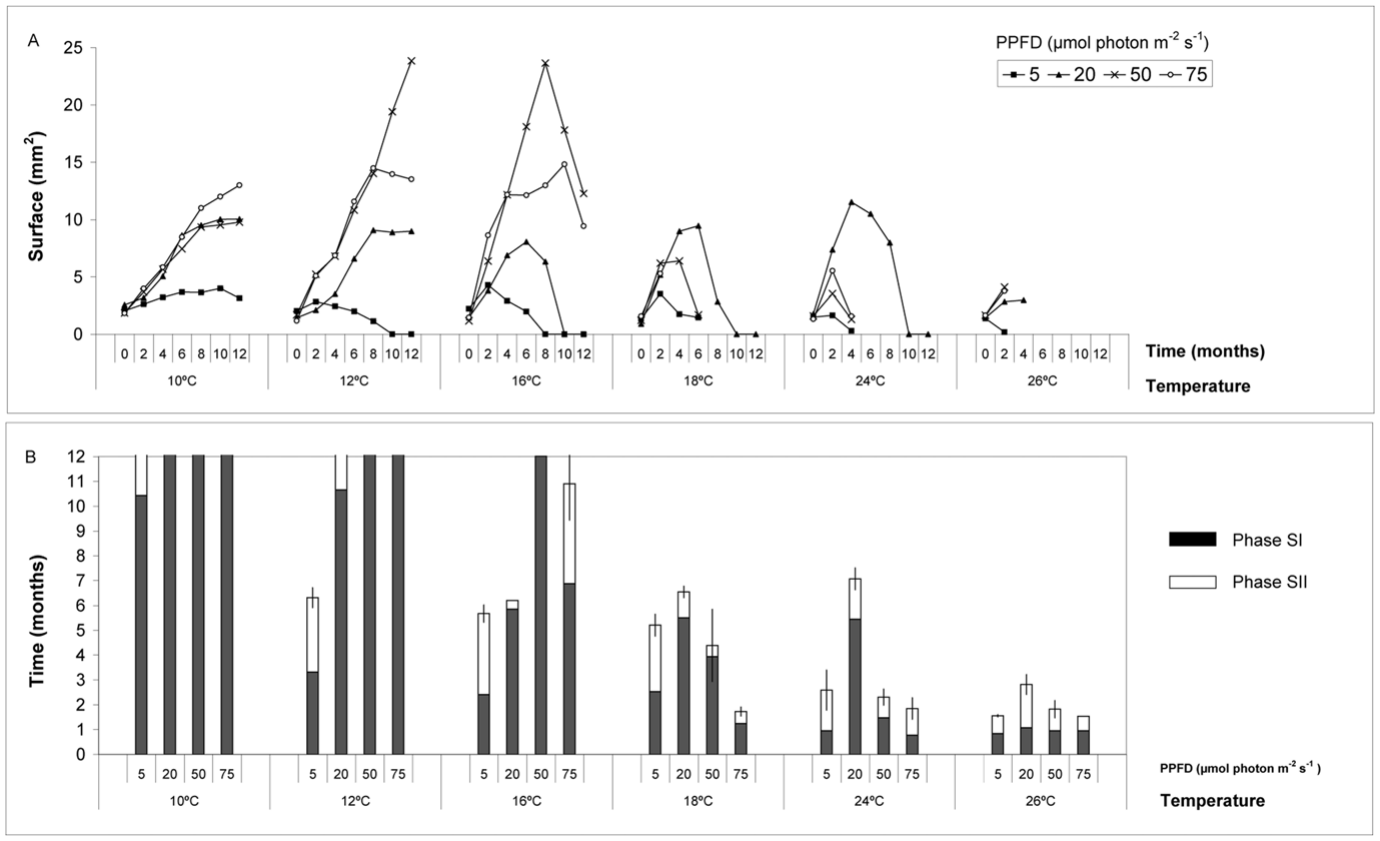
Ecophysiological experiments. A) Thallus surface of *W. setacea* along the culture period at the different PPFD assayed. B) Survival phases of *W. setacea* as a function of PPFD and temperature. Bars represent standard errors.

At the beginning of the experiment, all cultured specimens had a similar initial thallus surface (Fig. 5A; ANOVA-test, p>0.05). In the short term (one month), *W. setacea* was able to survive in all the combinations of temperature and PPFD assays, whereas in the mid term (3 months) most specimens cultured at high temperatures died, and in the long term (1 year) only those cultured below 18°C survived (Figs. 5A, 5B).

**Figure 6:**
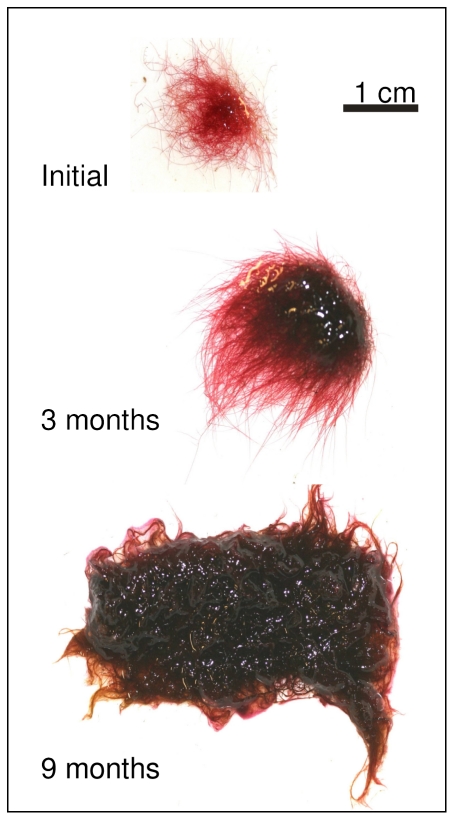
*Womersleyella setacea* growth during the ecophysiological experiments. The same specimen at the initial time, after 3 and 9 month of culture.

*Womersleyella setacea* survival was both temperature and PPFD dependent (Table 3, Fig. 5B), surviving in Phase SI during 1 year only at 10°C combined with 5–75 µmol photon m^−2^ s^−1^, at 12°C combined with 20–75 µmol photon m^−2^ s^−1^, and at 16°C combined with 50 µmol photon m^−2^ s^−1^ (Fig. 5B). The optimal conditions were very different in the short, mid and long term. At short and mid term, the optimal conditions were 16°C combined with 50–75 µmol photon m^−2^ s^−1^, but at the end of cultures (1 year), the specimens that still were in Phase SI, looked healthier and continued to maintain an increase in thallus surface were those cultured at 10°C and 20–75 µmol photon m^−2^ s^−1^ and at 12°C and 50 µmol photon m^−2^ s^−1^ (Fig. 5A). The maximum growth was observed in these last conditions (Figs. 5A, 6). Most specimens cultured at 24 and 26°C only persisted in Phase SI for less than 1 month (Fig. 5B).

**Table 3:** Results of the two-way ANOVAs on the survival of *W. setacea* and on the length of the Phase SI with temperature and light as fixed factors.

	df	MS	F	p
Survival				
Temperature	5	10803.5	531.00	<0.001
PPFD	3	1462.4	71.88	<0.001
Temperature×PPFD	15	695.7	34.20	<0.001
Error	72	20.3		
Length of Phase SI				
Temperature	5	10876.6	248.01	<0.001
PPFD	3	2131.8	48.65	<0.001
Temperature×PPFD	15	590.1	13.48	<0.001
Error	72	43.8		

### Phenology

We did not observe any reproductive structure (sporangia or gametangia), either in the field or in the cultured specimens.

## Discussion

The present study provides data on the abundance and temporal variability of *W. setacea* in the field. We observed high values of *W. setacea* biomass (up to 126 g dw m^−2^ in November at Imbutu), which are of the same order of magnitude as those recorded in other Mediterranean areas [5]. Similarly, the values obtained are comparable to those of other well-known invasive species thriving in benthic Mediterranean assemblages. For instance, values reported for the Chlorophyta *Caulerpa racemosa*, although they can be much higher, rarely exceed 100 g dw m^−2^ [30]–[33], and those reported for the Rhodophyta *Lophocladia lallemandii* range between 50 and 200 g dw m^−2^ [34]. Similarly, although maximum values of *Caulerpa taxifolia* in Mediterranean rocky bottoms range between 200 and 500 g dw m^−2^ [35]–[37], most *C. taxifolia* populations exhibit lower biomass values (from 50 to 200 g dw m^−2^) [38]–[41].

In general, no seasonal variation pattern has been found in the Scandola MPA populations of *W. setacea*. A dense thick red filamentous turf was widespread and persistent throughout the year at both localities studied, except in April, when biomass showed a slight decrease, in agreement with previous studies on *W. setacea* populations in other Mediterranean areas [5], [9], [13]. Populations of *W. setacea* from the Scandola MPA seem to propagate only by vegetative ways, what also agrees with other observations available for other Mediterranean regions in the field [5] or in cultures [24]. However, the presence of tetrasporangia was reported in the original collections from Hawaiian populations [16].

Mediterranean *W. setacea* is considered to be a sciaphilic species constituting thick carpets in deep waters [5]. However, its bathymetric distribution may vary in different areas, probably in function of the environmental features of each region (e.g., in Greece it was observed between 10 and 20 m depth [6], in the northern Aegean Sea between 15 and 30 m depth [13], in the Adriatic Sea, France and Italy between 10 and 30 m depth [5], [9], [18], and in the Spanish coast between 15 and 30 m depth (Cebrian, pers. obs.)). The bathymetric distribution of *W. setacea* shows that in the Scandola MPA it is restricted to depths between 25 and 35 m.

The present light requirements confirm the sciaphilic behavior of *W. setacea*, which presents a maximal survival and growth at PPFD ranging between 10 and 75 µmol photon m^−2^ s^−1^. Depth limits of algal distributions at increasing depths are related to the decrease of survival at lower PPFD [42]–[44]. Lower survival and growth of *W. setacea* were observed at lower PPFD (5 µmol photon m^−2^ s^−1^), explaining that in the Scandola MPA *W. setacea* almost disappeared or, at least, did not form dense carpets below 40 m depth, where PPFD were usually below 10 µmol photon m^−2^ s^−1^.

**Figure 7:**
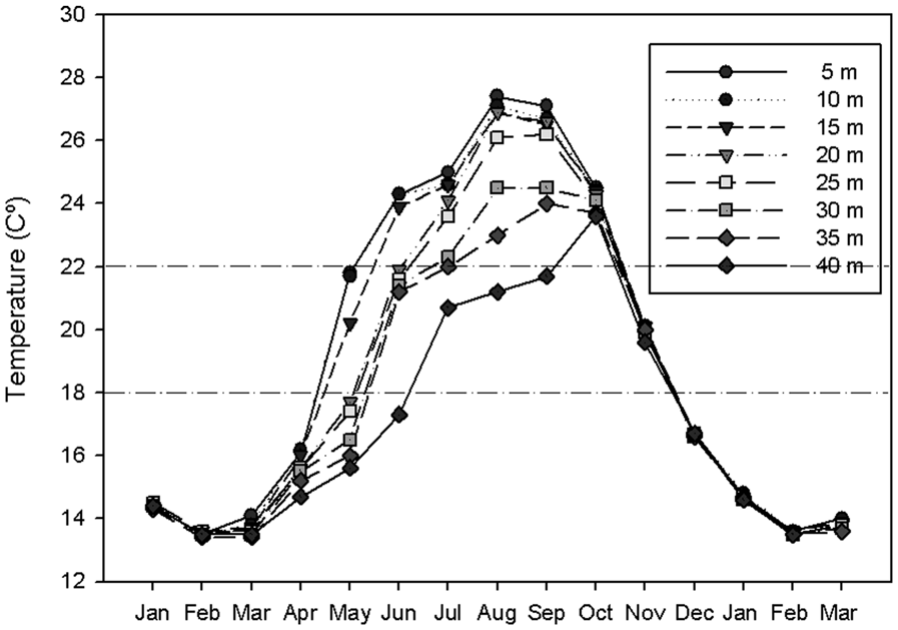
Mean monthly temperature data from SOMLIT (temperature sensors) data series located *in situ* at 5, 10, 15, 20, 25, 30, 35 and 40 m depth.

Results show that Mediterranean populations of *W. setacea* are adapted to relatively cold waters, specifically below 16°C. In the western Mediterranean these temperature conditions are common in the entire water column in winter and spring but are restricted to below the thermocline in summer and autumn (Fig. 7), at depths also characterized by low light conditions. In fact, all the specimens submitted to a constant temperature of 10°C experienced a small but persistent growth during the entire culture period, explaining why the thick carpets of *W. setacea* are present all year long in the field, and why winter temperatures are not a limiting factor for *W. setacea* invasiveness. These clear temperate water affinities compromise the until now stated tropical origin of *W. setacea*, highlighting the need for further comparative molecular investigation on the phylogeny and biogeography of Mediterranean and extra-Mediterranean populations of *W. setacea* to clarify the relationship of the invasive strain to the original tropical strain. Tolerance to low temperatures also occurs in other invasive species dwelling in the Mediterranean. For instance, the invasive Mediterranean strain of *Caulerpa taxifolia* is able to survive three months at temperatures ranging from 10 to15°C [45]–[46]. Nevertheless, the Mediterranean strain of *W. setacea* differs from *C. taxifolia* in its inability to support warm temperatures. *W. setacea* would be able to survive summer periods (of less than two months) in the upper infralittoral only at 20 µmol photon m^−2^ s^−1^ combined with temperatures ≤24°C. This explains in part the absence of *W. setacea* at the shallow infralittoral algal assemblages in the Scandola MPA, where temperatures commonly exceed 24°C (Fig. 7). Similarly, temperature requirements support the fact that in all Mediterranean sites where *W. setacea* has been reported it never develops thick carpets above 10 m depth, where temperatures can rise up to 25–30°C ([5]–[6], [9], [18,], this work).

The lack of seasonality of *W. setacea* biomass in the field is in agreement with the seasonal experiments at the laboratory, where no differences were found in the surface increase among the different periods of the year, probably because seasonality of underwater PPFD, temperature, hydrodynamics, and nutrient availability are minimized with depth [47]–[50]. Besides, *W. setacea* is capable of continuous asexual vegetative spread throughout the year [6], [24], [51], creating a very stable and homogenous habitat which may contribute to the loss of seasonality in the macrobenthic community structure [13].

*Womersleyella setacea* can thrive as an epiphyte and is able to overgrow other sessile benthic organisms. Therefore it is not limited by the availability of free substrate, and avoids competition with natives for substrate. On the other hand, invulnerability to native herbivores may be an important determinant of invasion success of marine macroalgae. While some studies reject the biological resistance hypothesis for marine macroalgae (e.g. [52]–[53]), others provide evidence that invasive algae are actively grazed by native herbivores, which can control their populations [54]–[55]. However, recent research addressed whether native generalist herbivores may provide resistance to macroalgae invasion in the Mediterranean Sea reveal that *W. setacea* is not consumed either by sea urchins nor *Sarpa salpa* [56]–[58]. Thus, we can hypothesize that in absence of a biological resistance in the new habitat, the main factors driving *W. setacea* development are light, temperature, and probably physical disturbance. The importance of light and temperature as major factors determining seaweed distribution has been stressed by many authors (see review in [59]); likewise, our field observations and experimental work revealed these factors as being critical for *W. setacea* survival and growth, and they are probably determining the spread and bathymetric distribution of this species across the Mediterranean Sea.

The invasion success of *W. setacea* in Mediterranean deep water assemblages probably relies on two different abilities. The first one is the ability to maintain permanent carpets all year long, outcompeting both perennial and ephemeral native species. The second ability would be the enhanced and sustained growth of *W. setacea*, higher than those recorded for other Mediterranean perennial or pseudoperennial native macroalgae growing in deep waters and cultured under the same conditions [60]–[62]. Thus, high growth and persistence should be the basis of *Womersleyella setacea's* capacity to outcompete native and engineering macroalgae and invertebrates from deep-water Mediterranean bottoms [8], [15]. Additionally, *Womersleyella setacea* traps sediment and, in this way, it may pre-empt space and prevent the attachment of possible spatial competitors [9] as described for other filamentous species [63]. This makes the settlement of native species and the survival of their juvenile stages impossible [64], thus reducing the species diversity and equitability of phytobenthic communities [5], [7], [65]–[66].

In brief, populations of *W. setacea* thriving in Mediterranean coastal waters exhibit thermal features of a temperate seaweed rather than a tropical one. Thus, winter temperatures are not a limiting factor in the survival and spread of *W. setacea* all over the Mediterranean Sea, and hot summer temperatures prevent its distribution in shallow waters. *Womersleyella setacea* also performs much better at low light intensities around 50 µmol photon m^−2^ s^−1^, which is in accordance with the depth distribution found in the field. We suggest that the invasion success of *W. setacea* in Mediterranean environments relies on its high growth, persistence and resistance to herbivores, which outcompetes both perennial and ephemeral native species growing at the same depth intervals where *W. setacea* develops.

## References

[1] Parker IM, Simberloff D, Lonsdale WM, Goodell K, Wonham M (1999). Impact: toward a framework for understanding the ecological effects of invaders.. Biol Inv.

[2] Zenetos A, Gofas S, Verlaque M, Çinar ME, García Raso JE (2010). Alien species in the Mediterranean Sea by 2010. A contribution to the application of European Union's Marine Strategy Framework Directive (MSFD). Part I. Spatial distribution.. Med Mar Sci.

[3] Boudouresque CF, Verlaque M (2002). Biological pollution in the Mediterranean Sea: invasive versus introduced macrophytes.. Mar Poll Bull.

[4] Thresher RE, Pederson J (2000). Key threats from marine bioinvasions: a review of current and future issues.. Marine Bioinvasions.

[5] Airoldi L, Rindi F, Cinelli F (1995). Structure, seasonal dynamics and reproductive phenology of a filamentous turf assemblage on sediment influenced, rocky subtidal shore.. Bot Mar.

[6] Athanasiadis A (1997). North Aegean Marine Algae IV. *Womersleyella setacea* (Hollenberg) R.E. Norris (Rhodophyta, Ceramiales).. Bot Mar.

[7] Piazzi L, Cinelli F (2001). Distribution and dominance of two introduced turf-forming macroalgae on the coast of Tuscany, Italy, northwestern Mediterranean Sea in relation to different habitats and sedimentation.. Bot Mar.

[8] Ballesteros E (2006). Mediterranean coralligenous assemblages: a synthesis of the present knowledge.. Oceanography and Marine Biology: an Annual Review.

[9] Battelli C, Rindi F (2008). The extensive development of the turf-forming red alga *Womersleyella setacea* (Hollenberg) R. E. Norris (Rhodophyta, Ceramiales) in the Bay of Boka Kotroska, Montenegro (southern Adriatic Sea).. Plant Biosystems.

[10] Piazzi L, Pardi G, Balata D, Cecchi E, Cinelli F (2002). Seasonal dynamics of a subtidal north-western Mediterranean macroalgal community in relation to depth and substrate inclination.. Bot Mar.

[11] Serio D, Alongi G, Catra M, Cormaci M, Furnari G (2006). Changes in the benthic algal flora of Linosa Island (Straits of Sicily, Mediterranean Sea).. Bot Mar.

[12] Airoldi L (2000). Effects of disturbance, life histories, and overgrowth on coexistence of algal crusts and turfs.. Ecology.

[13] Antoniadou C, Chintiroglou C (2007). Zoobenthos associated with invasive red alga *Womersleyella setacea* (Rhodomelaceae) in the northern Aegean Sea.. J Mar Biol Ass UK.

[14] Piazzi L, Balata D (2009). Invasion of alien macroalgae in different Mediterranean habitats.. Biol Inv.

[15] Linares C, Cebrian E, Coma R (in press). Turf algae and Mediterranean gorgonian forests: assessing the effects of turf algae on gorgonian recruitment and juvenile survival.. Mar Ecol Prog Ser.

[16] Hollenberg GJ (1968). An account of the species of *Polysiphonia* of the central and western tropical Pacific.. Pacific Sci.

[17] Guiry MD, Guiry GM (2011). AlgaeBase.. http://www.algaebase.org.

[18] Verlaque M (1989). Contribution à la flore des algues de la Méditerranée: espèces rares ou nouvelles pour les côtes françaises.. Bot Mar.

[19] Benedetti-Cecchi L, Cinelli F (1989). Note on a *Polysiphonia* sp. (Rhodophyte, Ceramiales) collected at Rosignano Solvay (Western Mediterranean).. Giorn Bot Ital.

[20] Verlaque M (1994). Inventaire des plantes introduites en Méditerranée: origines et répercussions sur l'environnement et les activités humaines.. Oceanol Acta.

[21] Mack RN, Simberloff D, Lonsdale WM, Evans H, Clout M (2000). Biotic invasions: causes, epidemiology, global consequences, and control.. Ecol Appl.

[22] Theoharides KA, Dukes JS (2007). Plant invasion across space and time: factors affecting nonindigenous species success during four stages of invasion.. New Phytologist.

[23] Hewitt CL, Hayes KR, Leppäkoski E, Gollasch S, Olenin S (2002). Risk assessment of marine biological invasions.. Invasive Aquatic Species of Europe, Distribution, Impacts and Management.

[24] Rindi F, Guiry MD, Cinelli F (1999). Morphology and reproduction of the adventive Mediterranean rhodophyte *Polysiphonia setacea*.. Hydrobiologia.

[25] Cebrian E, Ballesteros E (2004). Zonation patterns of benthic communities in an upwelling area from the western Mediterranean (La Herradura, Alboran Sea).. Sci Mar.

[26] Sala E, Ballesteros E (1997). Partitioning of space and food resources by three fish genus *Diplodus* (Sparidae) in a Mediterranean rocky infralittoral ecosystem.. Mar Ecol Prog Ser.

[27] Guiry MD, Cunningham EM (1984). Photoperiodic and temperature responses in the reproduction of north-eastern Atlantic *Gigartina acicularis* (Rhodophyta, Gigartinales).. Phycologia.

[28] Lewin J (1966). Silicon metabolism in diatoms. V. Germanium dioxide, a specific inhibitor of diatoms growth.. Phycologia.

[29] Droop MR (1967). A procedure for routine purification of algal cultures with antibiotics.. Br Phycol Bull.

[30] Ruitton S, Verlaque M, Boudouresque CF (2005). Seasonal changes of the introduced *Caulerpa racemosa* var. *cylindracea* (Caulerpales, Chlorophyta) at the northwest limit of its Mediterranean range.. Aquat Bot.

[31] Mezgui Y, Djellouli AS, Ben Chikh Almi I, Pergent-Martini C, El Asmi S (2007). Étude biométrique (biomasse et phénologie) des populations à *Caulerpa racemosa* dans la région de Bizerte (Tunisie)..

[32] Klein J, Verlaque M (2008). The *Caulerpa racemosa* invasion: a critical review.. Mar Pollut Bull.

[33] Cebrian E, Ballesteros E (2009). Temporal and spatial variability in shallow- and deep-water population of the invasive *Caulerpa racemosa* var. *cylindracea* in the Western Mediterranean.. Est Coast Shelf Sci.

[34] Cebrian E, Ballesteros E (2010). Invasion of Mediterranean benthic assemblages by red alga *Lophocladia lallemandii* (Montagne) F. Schmitz: depth-related temporal variability in biomass and phenology.. Aquat Bot.

[35] Meinesz A, Benichou L, Blachier J, Komatsu T, Lemée R (1995). Variations in the structure, morphology and biomass of *Caulerpa taxifolia* in the Mediterranean Sea.. Bot Mar.

[36] Thibaut T, Meinesz A, Coquillard P (2004). Biomass seasonality of *Caulerpa taxifolia* in the Mediterranean Sea.. Aquat Bot.

[37] Iveŝa L, Devescovi M (2006). Seasonal vegetation patterns of the introduced *Caulerpa racemosa* (Caulerpales, Chlorophyta) in the northern Adriatic Sea (Vrsar, Croatia).. Period Biol.

[38] Garrigue C (1994). Biomasse et répartition de *Caulerpa taxifolia* dans les lagons de Nouvelle-Calédonie.. Oceanol Acta.

[39] Pillen TL, Ingeltaube P, Dennison WC, Tibbets IR, Hall NJ, Dennison, WC (1998). Are expanding populations of the tropical green alga *Caulerpa taxifolia* a potential threat for Moreton Bay?. Moreton Bay and Catchment.

[40] Williams SL, Grosholz ED (2002). Preliminary reports from the *Caulerpa taxifolia* invasion in southern California.. Mar Ecol Prog Ser.

[41] Iveŝa L, Jaklin A, Devescovi M (2006). Vegetation patterns and spontaneous regression of *Caulerpa taxifolia* (Vahl) C. Agardh in Malinska (Northern Adriatic, Croatia).. Aquat Bot.

[42] Drew EA, Earll R, Erwin DG (1983). Light.. Sublittoral ecology: the ecology of shallow sublittoral benthos.

[43] Dring MJ (1981). Chromatic adaptation of photosynthesis in benthic marine algae: an examination of its ecological significance using a theoretical model.. Limnol Oceanogr.

[44] Ramus J, van den Meer JP (1983). A ecophysiological test of the theory of complementary chromatic adaptation. I. Color mutants of a red seaweed.. J Phycol.

[45] Chisholmn JR, Marchioretti M, Jaubert JM (2000). Effect of low water temperature on metabolism and growth of a subtropical strain of *Caulerpa taxifolia* (Chlorophyta).. Mar Ecol Prog Ser.

[46] Komatsu T, Meinesz A, Buckles D (1997). Temperature and light responses of alga *Caulerpa taxifolia* introduced into the Mediterranean Sea.. Mar Ecol Progr Ser.

[47] Ballesteros E, Zabala M, Alcover JA, Ballesteros E, Fornós JJ (1983). El bentos: el marc físic.. Història Natural de l'arxipèlag de Cabrera.

[48] Ballesteros E (1992). Els vegetals i la zonació litoral: espècies, comunitats i factors que influeixen en la seva distribució.. I E C Arx Sec Ciènc.

[49] Garrabou J (1997). Structure and dynamics of north-western Mediterranean rocky benthic communities along a depth gradient: a Geographical Information System (GIS) approach..

[50] Zabala M, Ballesteros E (1989). Surface-dependent strategies and energy flux in benthic marine communities or, why corals do not exist in the Mediterranean.. Sci Mar.

[51] Hata H, Nishihira M (2002). Territorial damselfish enhances multi-species co-existence of foraminifera mediated by biotic habitat structuring.. J Exp Mar Biol Ecol.

[52] Sumi CBT, Scheibling RE (2005). Potential role of grazing by sea urchins (*Strongylocentrotus droebachiensis*) in regulating the invasive alga *Codium fragile* ssp. *tomentosoides* in Nova Scotia.. Mar Ecol Prog Ser.

[53] Gollan JR, Wright JT (2006). Limited grazing pressure by native herbivores on the invasive seaweed *Caulerpa taxifolia* in a temperate Australian estuary.. Mar Fresh Res.

[54] Scheibling RE, Lyons DA, Sumi CBT (2008). Grazing of the invasive alga *Codium fragile* ssp. *tomentosoides* by the common periwinkle *Littorina littorea*: effects of thallus size, age and condition.. J Exp Mar Biol Ecol.

[55] Strong JA, Maggs CA, Johnson MP (2009). The extent of grazing release from epiphytism for *Sargassum muticum* (Phaeophyceae) within the invaded range.. J Mar Biol Assoc UK.

[56] Cebrian E, Ballesteros E, Linares C, Tomas F (2011). Do native herbivores provide resistance to Mediterranean marine bioinvasions? A seaweed example.. Biol Inv.

[57] Tomas F, Box A, Terrados J (2011). Effect of invasive seaweeds on feeding preference and performance of a keystone Mediterranean herbivore.. Biol Inv.

[58] Tomas F, Cebrian E, Ballesteros E (2011). Differential herbivory of invasive algae by native fish in the Mediterranean Sea.. Estuarine Coast Shelf Sci.

[59] Breeman AM (1988). Relative importance of temperature and other factors in determining geographic boundaries in seaweeds: experimental and phonological evidence.. Helgol Meeresunters.

[60] Izquierdo Ramírez C (2003). Morfologia, desenvolupament i adaptacions ecofisiològiques de dues poblacions de Rhodymenia ardissonei (Rhodymeniales, Rhodophyta) de la costa catalana en relació a la seva distribució batimètrica..

[61] Rodríguez-Prieto C, Joher S, Lara E, Pólo L, Sánchez N (2005). Requerimientos fisiológicos de luz, temperatura y fotoperíodo para el crecimiento de *Sebdenia rodrigueziana* (Halymeniales, Rhodophyta): primeros resultados..

[62] Sánchez N (2005). Estudi taxonòmic i ecofisiològic dels membres de la familia Faucheaceae (Rhodymeniales, Rhodophyta) de la península Ibèrica i de les illes Balears..

[63] Littler DS, Littler MM, Bucher KE, Norris JN (1989).

[64] Ballesteros E, Garrabou J, Hereu B, Zabala M, Cebrian E (2009). Deep-water stands of *Cystoseira zosteroides* (Fucales, Phaeophyta) in the Northwestern Mediterranean: insights into assemblage structure and population dynamics.. Estuarine, Coastal and Shelf Sci.

[65] Airoldi L, Virgilio M (1998). Responses of turf-forming algae to spatial variations in the deposition of sediments.. Mar Ecol Prog Ser.

[66] Morand P, Briand X (1996). Excessive growth of macroalgae: A symptom of environmental disturbance.. Bot Mar.

